# Mean platelet volume might be an effective indicator of poor semen quality in varicocele patients

**DOI:** 10.1007/s11255-024-04089-3

**Published:** 2024-05-24

**Authors:** Yangyang Mei, Pinpeng Xie, Dalu Liu, Bo Zhang, Xingliang Feng

**Affiliations:** 1 Department of Urology, Jiangyin People’s Hospital of Jiangsu Province, Jiangyin, China; 2 Department of Clinical Medicine, The Second School of Clinical Medicine, Anhui Medical University, Anhui, China; 3 Department of Urology, the First Affiliated Hospital of Anhui Medical University, Hefei, Anhui Province China; 4 Department of General Surgery, The Affiliated Chuzhou Hospital of Anhui Medical University, Chuzhou, Anhui China; 5 Department of Urology, The Third Affiliated Hospital of Soochow University, Changzhou, Jiangsu China

**Keywords:** Mean platelet volume, Platelet, Varicocele, Poor semen quality, Infertility

## Abstract

**Objective:**

To delve into the relationship between mean platelet volume (MPV) and semen quality in patients with varicocele.

**Materials and methods:**

A total of 246 varicocele patients and 120 healthy adult males were enrolled. Physical examinations and the color Doppler ultrasonography were conducted on patients with varicocele to confirm the diagnosis. Venous blood samples and semen samples were collected from all participants for subsequent analysis. A series of statistical analyses were conducted to assess the relationship between their MPV levels and semen quality. A series of statistical analyses were performed to assess the relationship between MPV and semen quality.

**Results:**

No statistically significant differences were found between body mass index (BMI), sexual hormones, semen volume, platelet count, and right testicular volume in all three groups (health subjects, varicocele without symptoms, and varicocele with infertility). When conducting regression analysis on two groups with varicocele, the results indicated that a lower MPV is associated with a reduced risk of varicocele accompanied by infertility (OR = 0.557 95% CI: 0.432–0.719, *P* < 0.001). Further correlation analysis in varicocele patients revealed that high MPV had a statistically negative impact on the occurrence of poor semen quality, affecting sperm concentration, progressive motility, and morphology (all *P* < 0.001). More importantly, when predicting varicocele associated with infertility, MPV demonstrated high diagnostic sensitivity (AUC = 0.745, *P* < 0.001).

**Conclusion:**

Our results indicate that MPV is higher in varicocele with infertility and is closely related to semen quality, which may suggest an accompanying decline in semen quality associated with varicocele. However, these conclusions require further experimental validation.

## Introduction

Varicocele is recognized to be the most common surgically treatable vascular cause of infertility, manifesting as dilatation and tortuosity of the spermatic plexus, which can be graded on palpation [1, 2]. Varicocele accounts for approximately 15–20% of the general male population, and its prevalence is higher in infertile men (approximately 35–40%) [3]. Varicocele accounts for approximately 15–20% of the male population and the prevalence is higher in infertile men (approximately 35–40%) [4]. In addition, varicocele may not only lead to pain, infertility, and even hypogonadism [5], but also increase the risk of hyperlipidemia, heart disease, and diabetes [6].

It is widely accepted that there are no exact parameters of semen that can be used directly to identify fertility, neither is there a generally accepted approximate model of fertility based on semen analysis [7]. Based on the fifth edition of the WHO reference range for semen analysis and the relevant literature, we know that sperm motility, sperm concentration, and normal morphology sperm are the three key indicators affecting semen quality [8–10]. Undoubtedly, infertility is one of the severe negative consequences caused by varicocele [11]. However, it is not true that all men with varicocele are infertile, and a large sample size study showed that 45–65% of the patients with varicocele (*n* = 1102) presented with normal seminal parameters [7]. Accordingly, the causality of the association between infertility and varicocele remains in some debate. It triggered a large number of scholars to study infertility associated with varicocele. Therefore, it is important to assess or predict semen quality in patients with varicocele by simple and convenient means in clinical practice.

In recent years, the platelets and their associated indices have been extensively examined in a number of vascular diseases. Indices of platelets such as platelet count (PCT), mean platelet volume (MPV), and platelet distribution width (PDW) represent markers indicating platelet function in the pathophysiology of the disease [12]. High MPV levels have been found to be associated with varicocele, which may present a possible risk factor for vascular endothelial lesions in varicocele patients [13–15]. However, there are some scholars taking the opposite view, who believe that there is no statistically significant difference in platelet indices between patients with varicocele and healthy controls [16]. In addition, a comparison between before and after surgical treatment of patients with varicocele combined with infertility has revealed that there is also an effect of MPV on semen quality in varicocele [17]. In our opinion, this is a worthy direction to investigate. We collected a larger sample size and tried to predict semen quality outcomes in patients with varicocele using MPV levels.

## Materials and methods

### Study population and study process

**Fig. 1:**
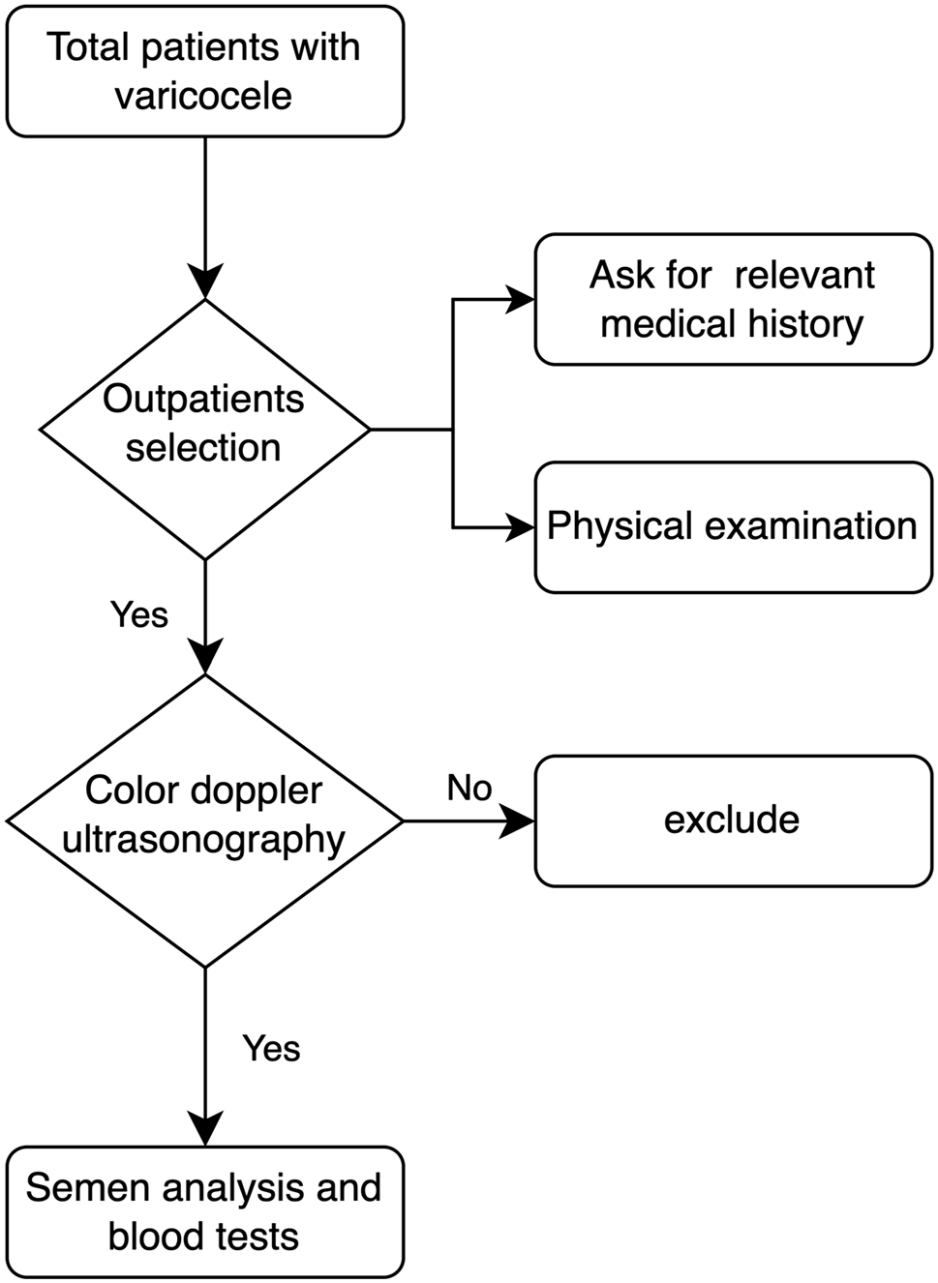
Simple process flow chart of the study

The prospective study was conducted in the Department of Urology and Andrology of the First Affiliated Hospital of Anhui Medical University between April 2019 and May 2021. We conducted this prospective study in accordance with the Declaration of Helsinki (as revised in 2013). The study was approved by the ethics committee of our hospital (PJ 2022-01-36). Informed consent for participation in the study has been obtained for all participants in a written format. All patients were consulted by the same Urological Surgeon at the First Affiliated Hospital of Anhui Medical University, and all patients were between the ages of 18 and 60. In the outpatient clinic, we took a detailed history of all patients, including previous and current physical status with varicocele, and performed a basic physical examination (including visual and palpation, under resting state and Valsalva maneuver). In addition, the following relevant medical histories were recorded: history of the conception, history of the scrotal disease, history of injury and surgery to the scrotum, groin, and perineum, history of alcohol consumption, history of smoking, history of medication, and history of systemic diseases: e.g., diabetes mellitus, hypertension, etc. Subsequently, color Doppler ultrasonography was performed for initial screening, and relevant findings were recorded. Finally, each patient was taken for routine blood tests and semen examination after 3–5 days of abstinence. A simple flow chart is shown in Fig. 1. A total of 246 varicocele patients were enrolled in our study from the clinic department, and another 120 healthy controls from the Healthy Physical Examination Center of our hospital were included in our study for comparison. Eventually, we divided all the participants into three groups named: Group 1 (varicocele with infertility); Group 2 (varicocele without symptom); Group 3 (healthy subjects).

## Inclusion and exclusion criteria

The diagnosis of varicocele was confirmed by physical examination and color Doppler ultrasonography. For inclusion criteria, different groups had different criteria. For Group 1, all patients should meet the following criteria: (1) age from 18 to 60 years old; (2) the initial diagnosis of left spermatic varicocele was made by physical examination and confirmed by ultrasonography; (3) active attempts to conceive for 1 year or more without success and diagnosed with infertility with at least two abnormal spermiogram parameters. Patients in Group 2 were recruited from young men preparing to join the army, and those with trouble preparing for pregnancy were excluded. Only patients who met the following criteria would be analyzed: (1) age from 18 to 60 years old; (2) the initial diagnosis of left spermatic varicocele was made by physical examination and confirmed by ultrasonography; (3) with normal spermiogram parameters (means that all parameters of semen analysis are above the limits of the WHO 5th edition reference range); (4) without any uncomfortable symptoms related to varicocele. For Group 3, all healthy subjects were men aged 18–60 years recruited from the Healthy Physical Examination Center of our hospital.

The exclusion criteria for all participants included: (1) history of scrotal disease other than varicocele (undescended testis, testicular torsion, epididymitis, orchitis, inguinal hernia, testicular mass); (2) history of surgery of the scrotum, perineum, or inguinal region; (3) with a systemic disease (coronary artery disease, diabetes mellitus, chronic renal failure, hematologic disease, hypertension, splenectomy, etc); (4) history of relevant medication use (including men taking any medications that may affect the hormonal axis (SERMs, testosterone, etc), medications that affect blood tests, and medications that may affect semen parameters, for instance, fluoroquinolones, cytostatic drugs, etc); (5) any high-risk lifestyle that may lead to blood and cardiovascular diseases, such as smoking, alcohol abuse, high-sodium diet.

## Physical examination and color Doppler ultrasonography

The physical examinations were conducted on all participants by a urological surgeon, and we would record varicocele side and degree of varicocele. First, the physical examination was conducted on participants without Valsalva to diagnose varicocele. Then the examination would be repeated with Valsalva to judge the degree of varicocele. Varicocele was diagnosed as three grades: Grade 1: varicocele could be examined with Valsalva; Grade 2: varicocele could be examined without Valsalva, but could not be seen with the eye; Grade 3: varicocele could be directly seen with the eye [18]. For varicocele patients, the color Doppler ultrasonography would be conducted for them to confirm the diagnosis. The color Doppler ultrasonography was conducted by a senior radiologist experienced in varicocele using an Aixplorer™ ultrasound system (Supersonic Imagine S.A., Aix-en-Provence, France). The spermatic vein diameter at rest (DR), spermatic vein diameter at Valsalva maneuver (DV), and the duration of reflux during the Valsalva maneuver were all measured by color Doppler ultrasound. Furthermore, the testis volume was also recorded by the ultrasound examination.

## Laboratory analysis

Venous blood samples were taken from the median cubital vein of all involved participants and placed in tubes containing ethylenediaminetetraacetic acid. All blood samples were analyzed within 2 h with SYSMEX XN-9000 hematology analyzer from Japan. The routine blood test of platelet included mean platelet volume (MPV) and platelet count (PLT). For semen analysis, the semen samples were obtained by masturbation, requiring sexual abstinence for 3–4 days. The semen samples were analyzed within 1 h after liquefaction at room temperature with SQA-V automated sperm quality analyzer from Israel according to WHO guidelines (2010). The primary parameters of semen analysis included semen volume, sperm progressive motility, sperm concentration, and sperm morphology.

## Statistical analysis

The statistical analyses were conducted using the SPSS statistical software version 25.0 (SPSS Inc., Chicago, IL, USA). For categorical variables, they were specified as proportions, and means (M) and standard deviation (SD) were used for continuous variables. The Chi-square and Fisher’s exact tests were used to compare the statistical differences between groups for categorical variables. For comparison of continuous variables between two groups, the student’s *T* test or the Mann–Whitney *U* test was used. One-way analysis of variance (ANOVA) was performed to compare the statistical difference of continuous variables between three groups when the variables were in accordance with normal distribution and homogeneity. Otherwise, the Kruskal–Wallis *H* test was performed. Univariate logistic regression and multivariate logistic regression were conducted to determine the significant predictor of varicocele with poor semen quality. Models were compared using the likelihood ratio test with and without corresponding variables. The Spearman correlation analyses were conducted to evaluate the correlation between MPV and semen parameters (semen volume, sperm concentration, sperm progressive motility, sperm morphology). Receiver operator characteristic (ROC) curves were used to calculate the cutoff value of the corresponding variable to differentiate varicocele patients with infertility and varicocele patients without symptoms. The area under the curve (AUC), sensitivity, and specificity of corresponding variables were also calculated. The statistical difference for all tests was set at a two-sided *P* < 0.05.

## Results

In the cohort study, we collected 246 patients with varicocele according to inclusion and exclusion criteria in outpatients (Group 1: 132 varicocele patients with infertility; Group 2: 114 patients diagnosed with varicocele due to an army medical examination) and we collected 120 healthy adult men at the physical examination center as our control group (Group 3). We collected basic information and data on physical examination, blood count, ultrasonography, and semen examination of all subjects, as detailed in Table 1. No statistically significant differences were found between BMI, semen volume, platelet count, and right testicular volume in all three groups after we performed the statistical methods of analysis described above. Only Groups 1 and 2 underwent physical and ultrasound examinations of the varicocele site. Therefore, comparisons of these parameters were conducted solely between these two groups. After the comparisons, we found statistically significant differences between the two groups in semen concentration, sperm progressive motility, sperm morphology, and MPV (all *P* < 0.001). Notably, all ultrasound findings (venous diameter, reflux time, and testicular volume) were not found to be statistically significantly different between the two groups. There was a significant difference in both left testicular volume and MPV in the varicocele group compared to healthy controls (*P* < 0.05). Regarding semen analysis results, no significant differences were found between Group 2 and Group 3. However, Group 1 displayed a statistically significant reduction in semen quality when compared to healthy subjects.

**Table 1:** Demographic and clinical characteristics of samples

	Group 1 (varicocele with infertility)	Group 2 (varicocele without symptom)	Group 3 (healthy subjects)	*P* value
Number,	132	114	120	–
Age, year	27.80 ± 4.55^**ψ**^	22.00 ± 1.81	27.43 ± 6.43	** < 0.001**^**a**^
BMI, kg/m^2^	22.19 ± 2.13	22.12 ± 2.47	22.65 ± 3.10	0.229^a^
Grade				
Grade I	35	28	–	0.970^c^
Grade II	64	57	–	
Grade III	33	32	–	
Spermiogram values				
Semen volume (ml)	3.28 ± 0.92	3.19 ± 0.88	3.17 ± 0.89	0.560^b^
Sperm concentration (10^6^/ml)	12.84 ± 5.20^**ϑξ**^	25.94 ± 5.99^**ζ**^	25.46 ± 6.93	** < 0.001**^**a**^
Sperm progressive motility (%)	17.38 ± 5.85^**ϑξ**^	36.10 ± 11.07^**ζ**^	37.14 ± 5.63	** < 0.001**^**a**^
Sperm morphology (%)	3.65 ± 1.65^**ϑξ**^	7.13 ± 1.64^**ζ**^	7.45 ± 1.25	** < 0.001**^**a**^
Platelet indices				
PLT, (10^9^/L)	227.39 ± 48.57	221.80 ± 46.01	224.74 ± 46.77	0.652^b^
MPV, (fL)	10.60 ± 1.79^**ϑξ**^	9.33 ± 1.78^**ω**^	8.88 ± 1.42	** < 0.001**^**a**^
Ultrasound parameters				
DR, mm	2.27 ± 0.75	2.18 ± 0.84	–	0.397^b^
DV, mm	2.41 ± 0.67	2.51 ± 0.71	–	0.223^b^
Reflux time, s	2.88 ± 1.38	2.94 ± 1.45	–	0.726^b^
Left testicular volume, (cm^3^)	18.75 ± 1.33^**ϑΦ**^	18.90 ± 1.40^**ω**^	19.62 ± 2.19	** < 0.001**^**a**^
Right testicular volume, (cm^3^)	19.59 ± 1.68	19.62 ± 2.02	19.68 ± 1.81	0.918^a^

Bold means the *p* value <0.001

^**ψ**^*P*: *P* > 0.05 when compared between Group 1 and Group 3

^**ϑ**^*P*: *P* < 0.05 when compared between Group 1 and Group 3

^**ξ**^*P*: *P* < 0.05 when compared between Group 1 and Group 2

^**ζ**^*P*: *P* > 0.05 when compared between Group 2 and Group 3

^**ω**^*P*: *P* < 0.05 when compared between Group 2 and Group 3

^**Φ**^*P*: *P* > 0.05 when compared between Group 1 and Group 2

^a^*P*: compared using one-way ANOVA

^b^*P*: compared using independent samples *t* test

^c^*P*: compared using Chi-square test

*BMI* body mass index, *PLT* platelet, *MPV* mean platelet volume, DR diameter at rest, *DV* diameter at Valsalva

**Fig. 2:**
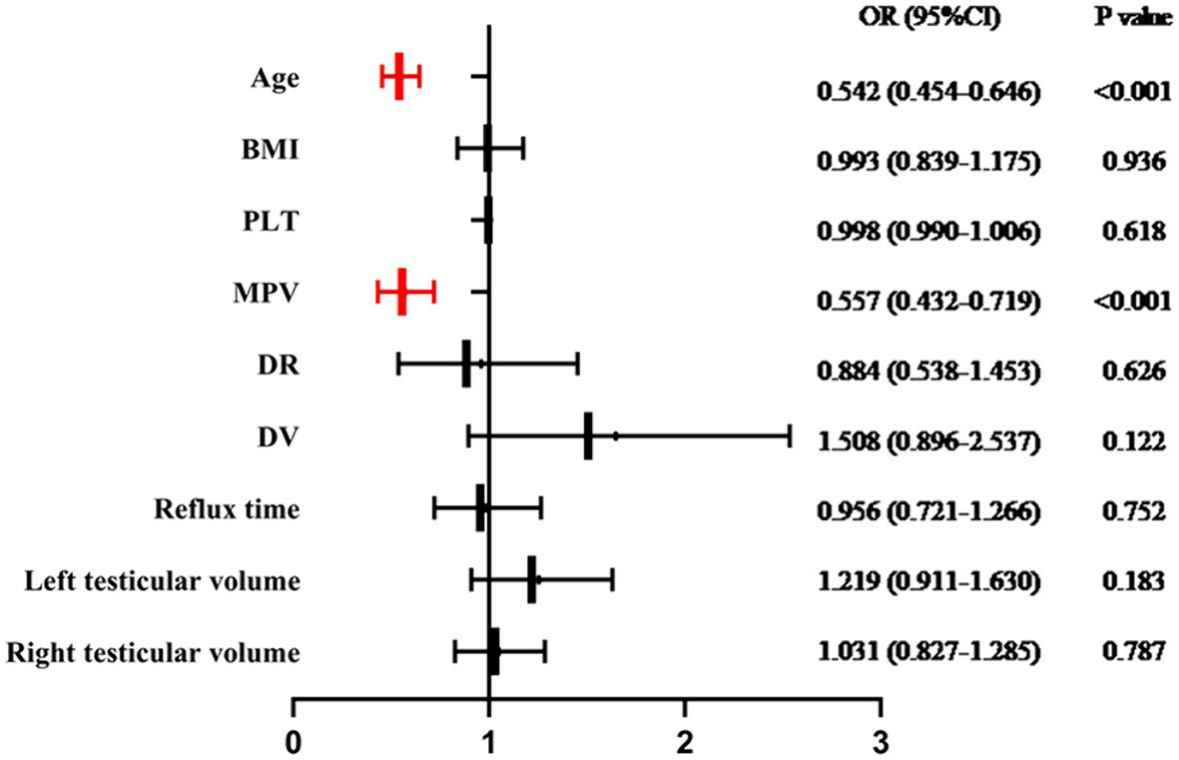
Multivariate logistic regression analyses of risk factors for varicocele related infertility

We further performed univariate logistic regression and multivariate logistic regression to analyze the factors contributing to the causes of poor semen quality among patients with varicocele. In the univariate regression analysis, both age (OR = 0.559, 95% CI 0.477–0.653, *P* < 0.001) and MPV (OR = 0.584, 95% CI 0.483–0.705, *P* < 0.001) showed statistically significant effects on the association between varicocele and infertility. However, varicocele grade did not demonstrate clinical significance. That is, compared to grade I, grades II (OR = 0.977, 95% CI 0.483–1.975, *P* = 0.947) and III (OR = 1.050, 95% CI 0.566–1.946, *P* = 0.878) did not exhibit a significant impact on the association between varicocele and infertility. Furthermore, we conducted multivariate regression analysis adjusting for all potentially relevant variables identified in the univariate analysis. The results revealed that young age (OR = 0.527, 95% CI 0.437–0.636, *P* < 0.001) and low MPV (OR = 0.553, 95% CI 0.427–0.716, *P* < 0.001) exhibited a protective effect against infertility in patients with varicocele. In other words, higher MPV levels were associated with a significant adverse impact on the occurrence of infertility in patients with varicocele. While several other factors were not found to be statistically significant, as detailed in Table 2 and Fig. 2. Building on this, we conducted Spearman’s correlation analysis between MPV and semen parameters in varicocele patients (Table 3). The findings indicated that elevated MPV significantly correlated with poor semen quality in varicocele patients, affecting sperm concentration (rho value = − 0.262), sperm progressive motility (rho value = − 0.612), and sperm morphology (rho value = − 0.474) (rho value < 0.001) (all *P* < 0.001). However, it did not influence semen volume (rho value < 0.001) (*P* = 0.997).

**Table 2:** Univariate and multivariate regression analyses results (varicocele patients with infertility vs. varicocele patients without symptoms)

	Univariate analysis		Multivariate analysis	
	*P* value	OR (95% CI)	*P* value	OR (95% CI)
Age	< 0.001	0.558 (0.477–0.653)	** < 0.001**	**0.527 (0.437–0.636)**
BMI	0.815	0.987 (0.884–1.102)	0.784	0.976 (0.824–1.158)
Grade I	Ref	Ref	Ref	Ref
Grade II	0.947	0.977 (0.483–1.975)	0.483	1.522 (0.471–4.922)
Grade III	0.878	1.050 (0.566–1.946)	0.617	0.777 (0.289–2.088)
PLT	0.357	0.997 (0.992–1.003)	0.881	0.999 (0.991–1.008)
MPV	< 0.001	0.584 (0.483–0.705)	** < 0.001**	**0.553 (0.427–0.716)**
DR	0.395	0.871 (0.633–1.198)	0.898	0.966 (0.574–1.628)
DV	0.223	1.256 (0.870–1.813)	0.093	1.580 (0.927–2.692)
Reflux time	0.734	1.033 (0.864–1.234)	0.722	0.950 (0.717–1.259)
Left testicular volume	0.392	1.084 (0.901–1.304)	0.209	1.207 (0.900–1.619)
Right testicular volume	0.897	1.009 (0.880–1.157)	0.920	1.012 (0.805–1.217)

Bold means the *p* value <0.001

*BMI* body mass index, *PLT* platelet, *MPV* mean platelet volume, *DR* diameter at rest, *DV* diameter at Valsalva, *OR* odd ratios, *CI* confidence interval

**Table 3:** Correlation between MPV and spermiogram values (Spearman analysis)

	MPV	
	rho value	*P* value
Semen volume	< 0.001	0.997
Sperm concentration	− 0.262	** < 0.001**
Sperm progressive motility (%)	− 0.612	** < 0.001**
Sperm morphology (%)	− 0.474	** < 0.001**

Bold means the *p* value <0.001

*MPV* mean platelet volume

**Fig. 3:**
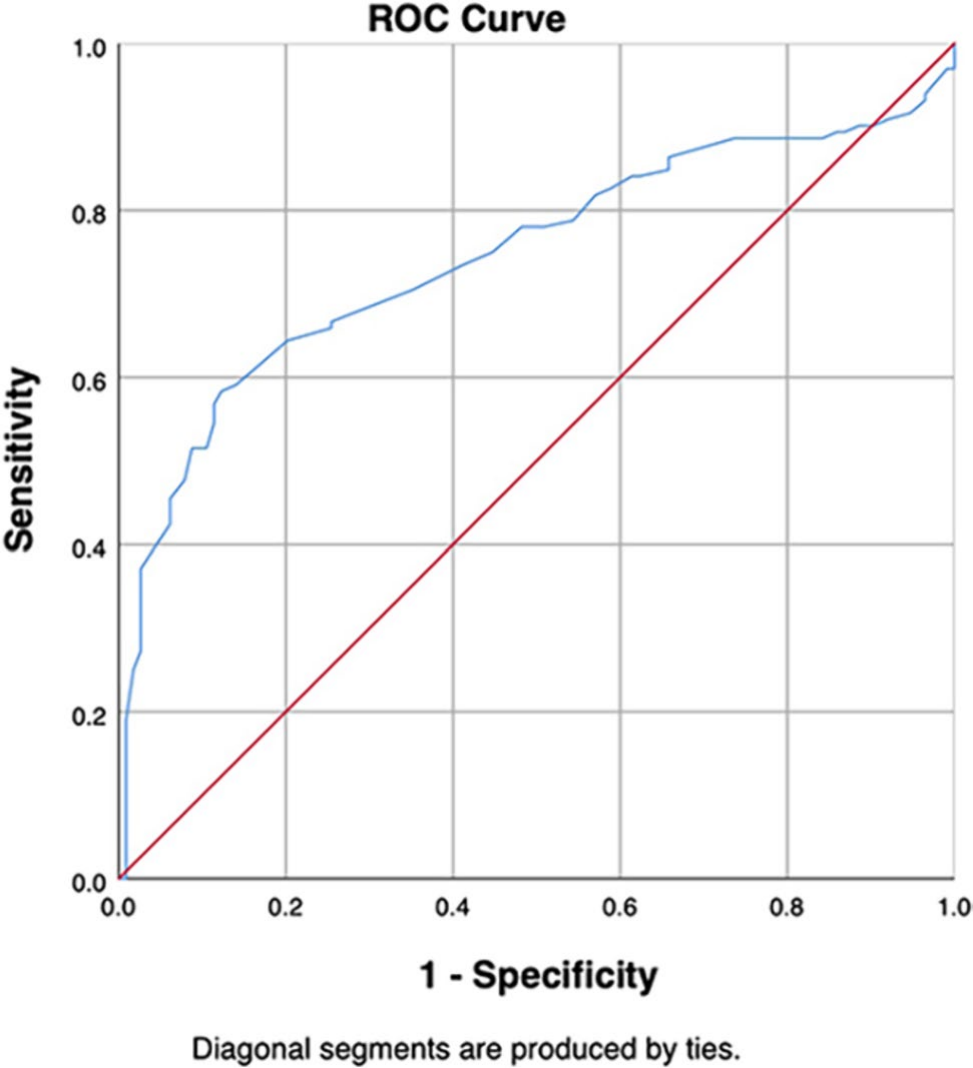
Receiver operator characteristic (ROC) curve of MPV in differentiating varicocele patients with infertility vs. varicocele without symptoms

In addition, we have depicted the ROC curve to evaluate the diagnostic value of MPV as an indicator to differentiate varicocele with infertility from varicocele without symptoms. The results showed that the general curve was to the upper left, and the overall analysis showed that MPV had a statistically significant diagnostic value to differentiate between the above two groups of patients with varicocele (AUC = 0.745, 95% CI 0.682–0.808, *P* < 0.001) with the cutoff value 10.35. In other words, when varicocele patients exhibit elevated MPV level (> 10.35), there is a higher likelihood of concomitant testicular damage and infertility, demonstrating good diagnostic sensitivity (0.583) and better specificity (0.877). The statistical results and curve graphs are shown in Fig. 3 and Table 4.

**Table 4:** ROC analysis of MPV as differentiating predictor between varicocele patients with infertility and varicocele patients without symptoms

	Cut-off value	AUC	95% CI	Sensitivity	Specificity	*P* value
MPV	10.35	0.745	0.682–0.808	0.583	0.877	< 0.001

*MPV* mean platelet volume, *AUC* area under curve, *CI* confidence interval

## Discussion

While the pathogenesis of varicocele remains incompletely understood, it is generally accepted that it may be related to a few factors that contribute to increased pressure in the seminiferous plexus or blood reflux [19, 20]. Generally, these factors include endothelial dysfunction, absence or insufficiency of the spermatic vein valve, the left spermatic vein flows vertically into the left renal vein, resulting in increased pressure in the spermatic plexus, or the left renal vein being pressed between the abdominal aorta and superior mesenteric artery which called “nutcracker effect” [21–23]. In addition, some scholars have found that higher MPV in patients with varicocele is related to graded varicocele increased [13, 14, 24]; however, there are some scholars who hold a contrary [16]. We evaluated the relationship between varicocele and these factors by age, BMI, MPV, semen analysis, and ultrasound examinations. Through our rigorous analysis, we concluded that: (1) the levels of MPV in patients with varicocele were statistically higher than controls (Group 1: *P* < 0.001, Group2: *P* = 0.009, combined Group 1 and Group 2: *P* < 0.001); (2) patients with varicocele combined with infertility presented statistically higher MPV levels than varicocele patients without symptoms; (3) left testicular volume was smaller in patients with left varicocele than in controls, but semen quality was not responsible for the decrease in testicular volume (*P* > 0.05). Interestingly, patients without semen quality problems were found to be younger in our study (*P* < 0.001), which is likely due to a bias caused by the young age of the population source of patients with varicocele found in the military medical examination.

Indeed, studies have demonstrated that MPV played a crucial role in several vascular disorders [25, 26]. Erectile dysfunction (ED) is one of the common conditions in urology, and a significant mechanism behind its onset is related to abnormalities in erection-associated blood vessels. Research has shown that MPV is closely linked to the onset of ED, effectively predicting the occurrence of arteriogenic ED, which is ED caused by arterial abnormalities [27]. These clinical studies confirm the clinical utility of MPV, but further research is needed to validate these findings and expand the application scope of MPV. This would help to better understand its potential across different vascular and systemic diseases, enhancing diagnostic, prognostic, and therapeutic strategies.

The first significant result was that MPV levels were statistically higher in patients with varicocele compared to the healthy subjects. As an essential component of blood, platelets were originally found to be capable of hemostasis and coagulation [28]. In recent years, the dynamic function of platelets has been studied extensively. The role of platelets in the etiology and pathogenesis of peripheral vascular, coronary, cerebrovascular, and other vascular diseases is gradually being unveiled [29]. MPV is measured by an automated hematology analyzer and commonly used to measure platelet activity [30]. In addition, it has been suggested that vascular endothelial injury may underlie the pathophysiology of spermatic varicose veins [31]. The high MPV value implies that larger platelets are more metabolically as well as enzymatically active and a tendency to aggregation, leading to thrombosis or endothelial dysfunction [32]. It motivated us to speculate on a possible association between MPV and varicocele, and it may provide inspiration and direction for further studies on the pathogenesis of varicocele and related vascular diseases in the future [13].

Activation of platelets plays an influential role in inflammation, which can be another underlying vascular cause of spermatic varicose veins. While higher MPV levels are thought to be an indicator of higher platelet activity, a series of cytokines, such as interleukin-6 (IL-6) and C-reactive protein (CRP), are generated in response to platelet activation [33]. McManus et al. have identified an important association between platelet gene expression and circulating inflammatory biomarkers such as C-reactive protein and IL-6, particularly in obesity and cardiovascular disease [34]. They suggested that varicocele was probably triggered by an inflammatory event and resulted in local and/or systemic inflammation. In addition, Demirer et al. reported that the increased MPV levels in varicocele patients probably are related to inflammation and the underlying mechanism may be the result of low-grade inflammation [35]. In conclusion, perhaps elevated MVP triggers local and/or systemic inflammation or endothelial dysfunction and ultimately affects the course of varicocele through the production of certain cytokines. Hence, MPV could potentially be used to predict varicocele, but this still requires more studies to confirm and reveal the intrinsic association between varicocele and MPV.

Another important result of our study was that in patients with varicocele, MPV levels were higher in patients with infertility than in patients without symptoms. The exact reasons why varicocele affects semen quality have not been revealed. However, a pilot meta-analysis in 2006 suggested that oxidative stress appears to play essential role in this complex pathophysiological process [36]. In addition, increased reactive oxygen species (ROS) levels increase with increasing grade of varicocele [37], and decrease after surgical treatment, as well as a consequent improvements in parameters of semen [38]. Oxidative stress can directly or indirectly injure germ cells by affecting non-spermatogenic cells and the basal lamina of the seminiferous tubules, thereby inducing apoptosis [39]. Besides, the imbalance between total antioxidant capacity (TAC) and ROS can contribute to the oxidation of fatty acids in sperm membranes, further leading to impairment of sperm motility, morphology, and fertilization [39]. It has been shown that platelet activation can lead to excessive production of ROS, which further induces oxidative stress, inflammatory response, and excessive platelet aggregation, contributing to cardiovascular events [40]. In addition, the occurrence of inflammation and the expression of inflammatory factors can contribute to some extent to testicular damage and affect semen quality [41].

Several studies have found that MPV can be used as an indicator to assess potential oxidative stress in various diseases such as cardiovascular disease, chronic obstructive pulmonary disease, and gestational diabetes, which can be further assessed for condition and prognosis [42–44]. MPV levels are also associated with the process and prognosis of many inflammatory events, which is an obvious factor in semen quality [45]. In conclusion, varicocele may contribute to infertility through multiple factors such as oxidative stress, inflammation, and testicular hypoxia. The increased MPV levels in patients who suffer from varicocele combined with infertility may result from negative factors such as inflammation and oxidative stress exacerbated by platelet activation.

In our cohort study, a statistically significant increased level of MPV was shown in patients with varicocele, with a further increase in patients combined with infertility. The ROC analysis revealed a cutoff value of 10.35, suggesting that patients with varicocele with MPV levels > 10.35 are likely to be accompanied by poor semen parameters. In our opinion, there is some clinical utility to this study: it could provide a clinical idea that MPV can be used as an indication to assess semen quality in patients with varicocele. Moreover, it is highly meaningful to monitor the MPV levels in routine blood tests during follow-up examinations after treatment to assess therapeutic outcomes. This can reflect the degree of inflammation in the body and potential changes in semen quality.

Another clinical significance of our study is the application of MPV in assessing semen quality. Varicocele adversely affects patients by causing discomfort and a decline in semen quality; the former can be easily identified through patient reports, while the latter often remains undetected due to its insidious nature [46]. Therefore, when infertility patients present with abnormally elevated MPV values, clinicians should consider the potential presence of varicocele, making further ultrasound and physical examinations crucial. Moreover, foundational research has utilized models of artificially induced varicocele to validate the application of MPV in the diagnosis of varicocele [47]. Our study provides direction for further basic research aimed at exploring the diagnostic value of MPV for varicocele associated with infertility.

On the other hand, several limitations should be recognized at the same time in our study. First, through the study, we showed a statistically significant correlation between MPV and infertility in patients with varicocele, but we could not further prove the precise mechanism. In the other words, it is possible that other factors contributing to infertility such as oxidative stress, inflammation, etc., contribute to the elevated MPV, which needs to be confirmed by further prospective cohort studies in the future. In addition, unilateral right sided and bilateral varicocele is excluded from our study. Therefore, our findings cannot be interpreted in patients with bilateral or right-sided spermatic varicoceles, which depends on the future expansion of the sample size for a more comprehensive study. Last but not least, the comparatively small quantity of patients in the study affected the power of the study, which needs to be confirmed by further studies with large sample sizes in the future.

## Conclusion

In summary, our study showed a statistically significant increase in MPV levels in patients with varicocele compared to healthy men, with a further increase in MPV levels in patients with combined infertility. It may suggest that a higher MPV level in varicocele patients could indicate an accompanying decline in semen quality associated with varicocele. However, further prospective cohort studies with larger sample sizes are needed to confirm the causal relationship between MPV and infertility in patients with varicocele.

## Data Availability

All data generated or analyzed during this study are included in this article. Further inquiries can be directed to the corresponding author.
